# Therapeutic potential of extracellular vesicles derived from *Platycladus Orientalis* leaf in treating anxiety, depression, and insomnia

**DOI:** 10.3389/fphar.2025.1693106

**Published:** 2025-09-29

**Authors:** Peiyu Liu, Guiyang He, Yaoxin Lu, Juanjuan He, Faliang Wu, Meng Jia, Shaochang Jia, Ruijie Fan, Hongwei Liang, Bing Liu, Min Li

**Affiliations:** ^1^ Department of Military Psychology, Army Medical University, Chongqing, China; ^2^ Lushan Rehabilitation and Sanatorium Center, Jiujiang, China; ^3^ Department of Emergency, Nanjing Drum Tower Hospital, School of Life Science and Technology, China Pharmaceutical University, Nanjing, China; ^4^ Institute of Geriatric Medicine, Jiangsu Province Geriatric Hospital, Nanjing, Jiangsu, China

**Keywords:** *platycladus orientalis (L.) franco*, extracellular vesicles, anxiety, depression, insomnia

## Abstract

**Objectives:**

Extracellular vesicles (EVs) mediate intercellular communication by transferring bioactive molecules. While animal-derived EVs are well studied, plant-derived EVs (plant-EVs) are emerging as stable, low-immunogenic nanocarriers with therapeutic potential. *Platycladus orientalis* (L.) Franco, used in traditional medicine, contains neuroactive compounds. This study evaluated the effects of *P. orientalis* leaf-derived EVs in rodent models of anxiety, depression, and insomnia.

**Methods:**

EVs were isolated by differential ultracentrifugation, characterized by electron microscopy and nanoparticle tracking analysis, and their bioactive components identified by GC -MS. Uptake was assessed in PC12 cells. Behavioral and biochemical effects were tested in mice subjected to chronic restraint stress (CRS), chronic unpredictable mild stress (CUMS), and para-chlorophenylalanine (PCPA)-induced insomnia. Key outcomes included social interaction, sucrose preference, Morris water maze performance, sleep parameters, neurotransmitter levels (5-HT, GABA), and inflammatory markers.

**Results:**

*P. orientalis* EVs exhibited bilayer vesicle morphology (∼100 nm) and contained abundant volatile compounds, particularly α-pinene. They were internalized by PC12 cells and reduced corticosterone-induced injury. In vivo, intranasal EV administration alleviated anxiety-like behaviors in CRS mice, restored sucrose preference and cognition in CUMS mice, and improved sleep onset and duration in PCPA-induced insomnia. Across models, EVs normalized serum and hippocampal 5-HT and GABA levels, reduced pro-inflammatory cytokines (TNF-α, IL-6), and increased TGF-β expression.

**Conclusion:**

*P. orientalis* leaf-derived EVs exert significant anxiolytic, antidepressant, and soporific effects through multimodal mechanisms involving neurotransmitter regulation and anti-inflammatory activity. Intranasal administration offers an effective strategy to bypass the blood -brain barrier, supporting the translational potential of plant-EVs as novel therapeutics for psychiatric disorders.

## Introduction

Psychiatric disorders, including anxiety, depression, and insomnia, represent a major global health challenge (Ustun and Chisholm, 2001; Palagini et al., 2024). Their progressive course and limited therapeutic options are compounded by complex disease mechanisms and the restrictive nature of the blood–brain barrier (BBB). While the BBB maintains brain homeostasis, it prevents the passage of over 98% of potential therapeutic agents, thereby impeding effective treatment. Addressing psychiatric disorders thus requires overcoming two critical challenges: enabling drug delivery across the BBB and targeting the multifactorial nature of disease. Conventional treatments rarely achieve both, underscoring the urgent need for novel strategies that combine efficient delivery with multimodal therapeutic effects.

Extracellular vesicles (EVs) are a heterogeneous group of cell-derived membranous structures that mediate intercellular communication by transferring bioactive molecules such as lipids, proteins, and RNAs between cells and tissues (Buck and Nolte-'t Hoen, 2024). These nanoscale vesicles, classified primarily as exosomes, microvesicles, migrasomes, and apoptotic bodies, play crucial roles in diverse biological processes, including immune regulation, tumor progression, and neuronal communication (Zhu et al., 2024; Li et al., 2025). More recently, plant-derived EVs have attracted increasing interest owing to their unique bioactive cargo, including secondary metabolites (e.g., flavonoids, polyphenols, and terpenoids) and small RNAs which are well known for their anti-inflammatory, antioxidant, and neuroprotective properties (Buck and Nolte-'t Hoen, 2024; Zhu et al., 2024; Li et al., 2025; Nueraihemaiti et al., 2025; Reed and Escayg, 2021; Zuo et al., 2025; Yu et al., 2025; Langellotto et al., 2025; Liu et al., 2024). Their natural origin, stability, and low immunogenicity make them attractive candidates for therapeutic applications. Previous studies have highlighted the ability of P-EVs to regulate inflammation, promote tissue repair, and modulate gut and systemic homeostasis, with most investigations focusing on oral or systemic delivery in cancer, intestinal, or metabolic models (e.g., ginger, grape, grapefruit, carrot) (Reed and Escayg, 2021; Yu et al., 2025; Isik et al., 2025). However, few studies have explored the potential of P-EVs in neuropsychiatric disorders. The blood–brain barrier (BBB) remains a major obstacle for central nervous system (CNS) drug delivery, and intranasal administration of EVs offers a direct and promising strategy to bypass this barrier and achieve nose-to-brain transport (Reed and Escayg, 2021; Yu et al., 2025; Zhang et al., 2025; Wang et al., 2025).

*Platycladus orientalis (L.) Franco (P. orientalis)*, a monoecious evergreen tree in the family Cupressaceae, is native to China and widely distributed across Asia and Europe, where it is one of the most common ornamental species (Wang et al., 2008). Its leaves have been used extensively in traditional East Asian medicine to treat ailments such as diabetes, the common cold, cough, bronchitis, asthma, rheumatoid arthritis, inflammatory disorders, and skin infections (Fan et al., 2012; Mostafa et al., 2024; Korany et al., 2025; Darwish et al., 2022; Cui et al., 2023; Fan et al., 2011; Zhang et al., 2016; Ren et al., 2017; Lin et al., 2016; Ren et al., 2019a; Ren et al., 2019b; Gan et al., 2021). These therapeutic effects are attributed to the presence of diverse bioactive compounds, including flavonoids, tannins, terpenoids, polysaccharides, and volatile oils, which confer antioxidant, antifungal, anti-inflammatory, antibacterial, diuretic, neuroprotective, detoxifying, and hair growth–promoting properties (Fan et al., 2012; Mostafa et al., 2024; Korany et al., 2025; Darwish et al., 2022; Cui et al., 2023; Fan et al., 2011; Zhang et al., 2016; Ren et al., 2017; Lin et al., 2016; Ren et al., 2019a; Ren et al., 2019b; Gan et al., 2021). Notably, clinical studies have demonstrated that *P. orientalis* leaf, used as the primary raw material in the traditional Chinese medicine formulation Naozhenning granules, can improve outcomes in traumatic brain injury (Cao et al., 2020). This evidence suggests that extracts of *P. orientalis* hold potential as therapeutic agents for psychiatric disorders.

To evaluate the therapeutic potential of *P. orientalis* leaf-derived EVs administered intranasally, we assessed their behavioral, biochemical, and molecular effects in three rodent models: chronic unpredictable mild stress (CUMS), chronic restraint stress (CRS), and para-chlorophenylalanine (PCPA)-induced insomnia. Our results demonstrated that *P. orientalis* leaf-derived EVs exert significant therapeutic effects in stress-related psychiatric disorders, highlighting their potential as a novel plant-based strategy for mental health management.

## Materials and methods

### Isolated EVs of *P. orientalis* leaf

EVs of *P. orientalis* leaf were isolated through differential ultracentrifugation from fresh leaves collected from Mount Lushan which was were authenticated by Lushan Botanical Garden, Jiangxi Province and Chinese Academy of Sciences. Initially, the leaves were crushed and blended into a liquid form. This mixture was then centrifuged at 4 °C for 30 min at 3,000 g to eliminuteuteuteate large cellular debris, followed by filtration using a 0.45 μm filter. Subsequently, the resulting supernatant was transferred to new ultracentrifuge tubes and subjected to another round of centrifugation at 10,000 g for 30 min at 4 °C. After removing the pellet, the supernatant was centrifuged again for 1 h at 120,000 g and 4 °C. The collected pellets were washed with 10 mL of infiltration buffer (20 mM MES hydrate, 2 mM CaCl2, 0.1M NaCl, pH 6.0), and then re-centrifuged at the same speed before being resuspended in infiltration buffer for further analysis.

### Analysis of EVs of *P. orientalis* leaf by transmission electron microscopy (TEM)

TEM was employed to examinuteuteutee the morphology and size of the EVs directly. The vesicles were first fixed in a solution containing 2.5% glutaraldehyde and 5% bovine serum albuminuteuteute (BSA), followed by postfixation with 2% osmium tetroxide. The samples underwent dehydration through a graded series of acetone and ethanol, then were embedded in epoxy resin (SPI Inc., Westchester, PA, United States). Ultrathin sections (80–90 nm) were sequentially stained with 5% uranyl acetate for 15 min, followed by 0.1% lead citrate for 5 min. Finally, electron micrographs were captured and analyzed using a Hitachi 7,500 transmission electron microscope.

### Analysis of EVs of *P. orientalis* leaf by nanoparticle tracking analysis (NTA)

NTA was carried out with the NanoSight NS300 system (Malvern, United Kingdom), which features a 488 nm laser and a high-sensitivity scientific CMOS (sCMOS) camera. The sample chamber temperature was maintained at 25 °C throughout the experiment. Measurements were taken under the following settings: camera level at 12, acquisition time of 30 s, and a detection threshold of 7. Particle movement was captured in triplicate, and the collected data were subsequently analyzed using NanoSight Software (version 2.3).

### Analysis of volatile components in *P. orientalis* leaf extracellular vesicles (EVs) by GC-MS

EVs from *P. orientalis* leaves were extracted by immersion in HPLC-grade n-hexane (SaFo Technology, Tianjin, China) within a sealed container for 24 h at room temperature. To enhance extraction efficiency, the samples were agitated periodically. Following extraction, a known concentration of ethyl decanoate (CAS 110-38–3, ≥98%; Sigma-Aldrich, St. Louis, MO, United States) was added to aliquots of the extract as an internal standard. These samples were then filtered through 0.22 µm nylon syringe filters prior to gas chromatography-mass spectrometry (GC-MS) analysis. Analysis was conducted using an Agilent 7890A-5975C GC-MS system (Agilent Technologies Inc., Santa Rosa, CA, United States) equipped with an HP-5 MS capillary column (30 m × 0.25 mm, 0.25 µm film thickness). A 1 µL sample was injected in splitless mode with an injector temperature of 250 °C. Helium (99.999% purity) served as the carrier gas at a constant flow rate of 1.0 mL/min. The oven temperature program was initialized at 50 °C (held for 2 min), ramped to 180 °C at 5 °C/min, then increased to 270 °C at 20 °C/min, and finally held at 270 °C for 5 min. Mass spectrometry was performed with an electron ionization (EI) source temperature of 230 °C and an ionization energy of 70 eV. The mass spectrometer was operated in full-scan mode, acquiring data over a mass range of 40–500amu. Tentative identification of separated constituents was achieved by comparing their mass spectra with entries in the NIST08 mass spectral library (National Institute of Standards and Technology, Gaithersburg, MD, United States) and by comparing their calculated retention indices (RIs) with values reported in the scientific literature.

### EVs of *P. orientalis* leaf uptake experiment

Incubate Dil (1,1′-Dioctadecyl-3,3,3′,3′-Tetramethylindocarbocyanine Perchlorate (‘DiI’; DiIC18(3))) (Beyotime, C1991S) with the isolated EVs of *P. orientalis* leaf in the dark at room temperature (RT) for 30 min, with gentle inversion every 5 min. Subsequently, the mixture was centrifuged at 4 °C for 30 min at 3,000 g, followed by filtration using a 0.45 μm filter. The resulting supernatant was then transferred to new ultracentrifuge tubes and subjected to another round of centrifugation at 10,000 g for 30 min at 4 °C. After removing the pellet, the supernatant was centrifuged again for 1 h at 120,000 g and 4 °C. The collected pellets (the Dil labeled EVs of *P. orientalis* leaf) were resuspended with infiltration buffer for further analysis. The Dil labeled EVs of *P. orientalis* leaf was incubated with PC12 cells (a cell line derived from a pheochromocytoma of the rat adrenal medulla) at a concentration of 10^8^ EVs/mL culture medium for 12 h. After incubation, the nuclei were counterstained with DAPI. The prepared samples were finally examinuteuteuteed under a confocal microscope (Olympus FV3000).

### Cell viability and LDH leakage

Cells were seeded into 96-well plates at a density of 10^5^ cells per well and subjected to the indicated treatments. The cells treated with 500 μM corticosterone, 500 μM corticosterone plus 5 μg/mL (about 10^9^ particles/mL) or 500 μM corticosterone plus 10 μg/mL (about 2x10^9^ particles/mL) for 24 h. Cell viability was assessed using the Cell Counting Kit-8 (APExBIO, United States). Culture supernatants were subsequently harvested and analyzed for cytotoxicity with the Lactic Dehydrogenase Release Assay Kit (C0016, Beyotime), following the manufacturer’s protocol.

### Animals

Adult male C57BL/6 mice (8-week-old) were obtained from GemPharmatech LLC (Nanjing, China). They were maintained on a 12/12 light cycle. Food and water were available *ad libitum*. EVs of *P. orientalis* leaf were suspended in sterile phosphate-buffered saline (PBS) at a concentration of 50 μg/μL (based on total exosomal protein). Adult male mice were placed in a supine position with the head tilted approximately 45°. EVs were administered intranasally using a micropipette. The procedure was repeated once daily. Control animals received an equal volume of PBS. All animal experiments were conducted in accordance with institutional guidelines and approved by the China Pharmaceutical University Animal Care and Use Committee.

### Chronic restraint stress (CRS) model

After a 3-day adaptation period, 30 mice designated for the CRS group were placed individually in ventilated plastic cylinders (3 cm in diameter) for 10 h each day (9:00–19:00) over 14, 21, or 28 consecutive days. Cylinder length was adjusted according to body size to ensure adequate restraint. These animals were randomly divided into three subgroups: untreated model, citalopram treatment (10 mg/kg by intragastric gavage), and EV treatment (10 μg/kg adminuteuteuteistered intranasally). Meanwhile, 10 control mice were subjected to food and water deprivation for the same duration to parallel the CRS exposure. Following each restraint session, all mice were returned to their cages with free access to food and water. Body weight (BW) was monitored throughout the study.

### Chronic unpredictable mild stress (CUMS) model

During the CUMS procedure, three stressors were selected at random each day from a panel of eight predefined conditions. The randomization sequence was generated by computer (MATLAB R2021a) to prevent repetition on consecutive days and to ensure that each stressor was applied with approximately equal frequency across the 28-day schedule. The stress paradigms included: (1) 24 h food deprivation, (2) 24 h water deprivation, (3) 2 h restraint in a 50-mL tube, (4) 10 minuteuteute tail clipping, (5) 24 h exposure to a damp cage, (6) 5 minuteuteute swimminuteuteuteg in 4 °C water, (7) 2 h exposure to a novel odor, and (8) 10 minuteuteute cage shaking. During this period, drug adminuteuteuteistration was conducted simultaneously: the blank and model groups received an equivalent volume of distilled water, while the treatment groups were given EVs intranasally at 10 μg/kg once daily.

### Establishment of parachlorophenylalanine (PCPA) induced insomnia model

After a 7-day adaptation period, 50 mice with comparable open-field test (OFT) scores, body weight, and sucrose water intake were selected. Among them, 40 animals received intraperitoneal injections of PCPA suspension (0.4 g/kg) once daily for two consecutive days at 8:00 a.m. Successful model establishment was confirmed by loss of circadian rhythm and elevated daytime activity. These mice were then randomly assigned to four groups: model, diazepam, and EVs at low (5 μg/kg) or high (10 μg/kg) doses. The remaining 10 mice, without modeling, were used as the normal control group. Diazepam, serving as the positive control, was adminuteuteuteistered by gavage at 1.2 mg/kg once per day for 21 days. EVs were delivered intranasally at 5 μg/kg or 10 μg/kg daily over the same period. Both the normal control and untreated model groups were given an equal volume of distilled water.

### Social interaction test (SI)

The social interaction (SI) test is a two-step behavioral assay widely used as a reliable measure of depression-like phenotypes. Behavioral data from both phases were automatically recorded under red-light illuminuteuteuteation using AniLab software (AniLab Software & Instruments, Ningbo, China). In the initial 150-s session, a C57BL/6J mouse was allowed to freely explore an open-field arena (45 × 45 × 45 cm) containing a wire-mesh cage (10 × 6 × 45 cm) positioned against one wall but without a social target. In the subsequent 150-s session, the same mouse was returned to the arena, this time with an unfamiliar CD1 male placed inside the cage. After each trial, the apparatus was carefully wiped with 70% ethanol to eliminuteuteuteate residual odors. The SI index was calculated as: 100 × (time spent in the interaction zone with the target)/(time spent in the interaction zone without the target). Based on these scores, defeated animals were classified as susceptible (interaction ratio <100) or resilient (interaction ratio ≥100).

### Sucrose preference test (SPT)

The sucrose preference test (SPT) was conducted to evaluate anhedonia following previously described methods (Li et al., 2018). In brief, mice were individually housed and allowed to habituate to a 1% sucrose solution for two consecutive days. After a 24 h period of food and water deprivation, animals were given access to two bottles—one containing 1% sucrose and the other plain water. During testing, bottle positions were alternated every 12 h to prevent side bias, and the weight of each bottle was measured. Sucrose preference was expressed as the percentage of sucrose intake relative to total fluid consumption.

### Morris water maze (MWM) test

The Morris water maze test was used to evaluate hippocampus-dependent spatial learning and memory in mice. Mice were placed in a circular pool (160 cm in diameter, 50 cm in height) filled with water maintained at 24–26 °C. The pool was divided into four quadrants (A, B, C, and D), with a hidden platform positioned 2 cm below the water surface in quadrant A. Each mouse was gently placed into the water facing the wall of the pool, and the escape latency (time taken to climb onto the platform) was recorded. If the platform was not located within 90 s, the animal was guided to the platform and allowed to remain there for 10 s, with the escape latency recorded as 90 s before repeating the trial. Two trials were conducted per day to assess short-term memory formation, and this procedure was continued for 3 consecutive days. After completion of the acquisition phase, the submerged platform was removed, and each mouse was released into the pool facing away from the previous platform location. Swimminuteuteuteg behavior was monitored for 300 s, and the time spent in quadrant A (target quadrant) was recorded as the measure of spatial memory retention.

### The pentobarbital sodium–induced sleep test

Mice were intraperitoneally injected with pentobarbital sodium solution at a dose of 40 mg/kg. Sleep latency and duration were recorded based on the disappearance of the righting reflex for more than 30 s.

### Sample collection

After completion of all behavioral tests, the anesthesia was induced by intraperitoneal injection of 2,2,2-tribromoethanol (350 mg/kg, Sigma-Aldrich, T48402) and the tissue was collected. The mouse was immobilized by holding the neck with the left hand while securing the body and limbs with the palm to prevent movement. Using scissors, the whiskers were trimmed, and the eyeballs were immediately enucleated to collect blood into 1.5-mL centrifuge tubes. The samples were carefully placed in a 4 °C refrigerator and kept overnight. On the following day, the blood samples were centrifuged at 3,500 r/minuteuteute for 15 min at 4 °C to separate serum. After blood collection, the mice were euthanized by cervical dislocation and decapitation. The heads were placed on ice, and the whole brain was carefully dissected using forceps. The brains were rinsed in ice-cold physiological saline to remove blood, blotted dry with filter paper, and the hippocampus and prefrontal cortex were rapidly isolated. Each brain region was transferred into pre-labeled 2-mL EP tubes, immediately frozen in liquid nitrogen, and then stored at −80 °C until further analysis. Quantitative determinuteuteuteation of 5-HT and GABO was performed using a commercially available ELISA kit (Wuhan MSK Biotechnology Co., Ltd., Wuhan, China) and with high-sensitivity (6.25 pg/mL) according to the manufacturer’s instructions.

### RT-qPCR

Total RNA was isolated with TRIzol Reagent (Invitrogen, United States) and dissolved in diethylpyrocarbonate (DEPC)-treated water following the manufacturer’s protocol. The concentration and purity of RNA were measured using a OneDrop-2000 spectrophotometer (NanoDrop Technologies). For mRNA quantification, reverse transcription quantitative PCR (RT-qPCR) was carried out in 96-well plates with the HiScript III All-in-One RT SuperMix Perfect for qPCR (Vazyme Biotech, R333-01) together with the Taq Pro Universal SYBR qPCR Master Mix (Vazyme Biotech, Q712-02). The primer sequences applied in this work are provided in Supplementary Table S1.

### Statistical analysis

Data are expressed as mean ± SEM and were processed with SPSS software version 18.0 (SPSS Inc., Chicago, IL, United States). Depending on the experimental design, comparisons between groups were assessed using unpaired Student’s t-tests, one-way or two-way ANOVA, or repeated-measures ANOVA. When significant differences were detected in ANOVA, Bonferroni post hoc multiple comparison tests were applied. A probability value of p < 0.05 was considered statistically significant.

## Results

### Characterization of EVs from *P. orientalis* leaf

**FIGURE 1:**
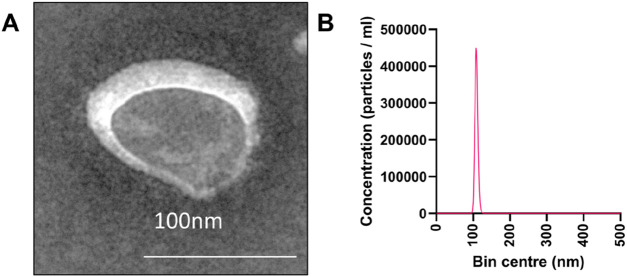
Isolation and characterization of EVs from *P. orientalis* leaf. **(A)** TEM analysis of EVs from *P. orientalis* leaf. The scale bar represents 100 nm. **(B)** NTA quantification of EVs from *P. orientalis* leaf.

EVs were isolated using differential ultracentrifugation and characterized by TEM and NTA. They exhibited the typical bilayer membrane structure and an average diameter of ∼100 nm (Figures 1A,B), consistent with previous reports (Xu M. et al., 2024). Approximately 10^12^ EVs were obtained per gram of leaves.

### Analysis of volatile components in EVs from *P. orientalis* leaf

**FIGURE 2:**
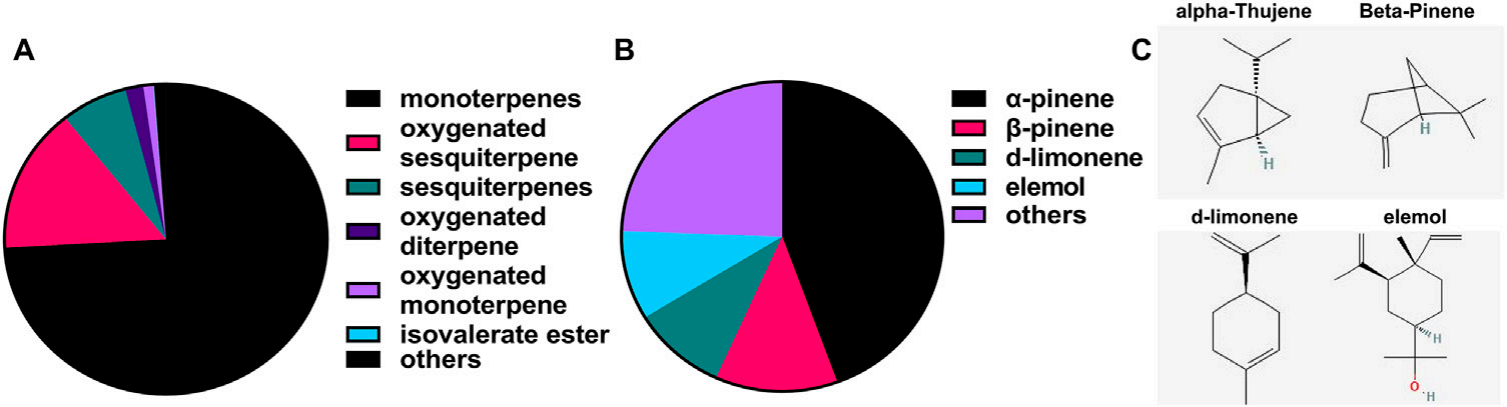
Volatile components in the EVs from P. orientalis leaf. **(A)** The Pie chart of the volatile components contained in EVs from *P. orientalis* leaf. **(B)** The Pie chart of the highest content of volatile components contained in EVs from *P. orientalis* leaf. **(C)** The structure of the top 4 highest content of volatile components contained in EVs from *P. orientalis* leaf.

The volatile components of EVs from *P. orientalis* leaf were analyzed using gas chromatography-mass spectrometry (GC-MS), which identified a total of 62 compounds. Consistent with the previously identified volatile components in *P. orientalis* leaf (Darwish et al., 2022; Hao et al., 2023; Shan et al., 2018), the major constituents were monoterpenes (15 compounds, 70.77%), followed by oxygenated sesquiterpenes (11 compounds, 16.22%), sesquiterpenes (20 compounds, 9.50%), oxygenated diterpenes (2 compounds, 1.97%), oxygenated monoterpenes (12 compounds, 1.81%), and isovalerate esters (2 compounds, 0.11%) (Figure 2A; Supplementary Table S2). Among these, α-pinene, β-pinene, d-limonene, and elemol were the most abundant components, with α-pinene accounting for more than 40% of the total volatile content (Figures 2B,C; Supplementary Table S2).

### Gene ontology (GO) and kyoto encyclopedia of genes and genomes (KEGG) pathway enrichment analysis

**FIGURE 3:**
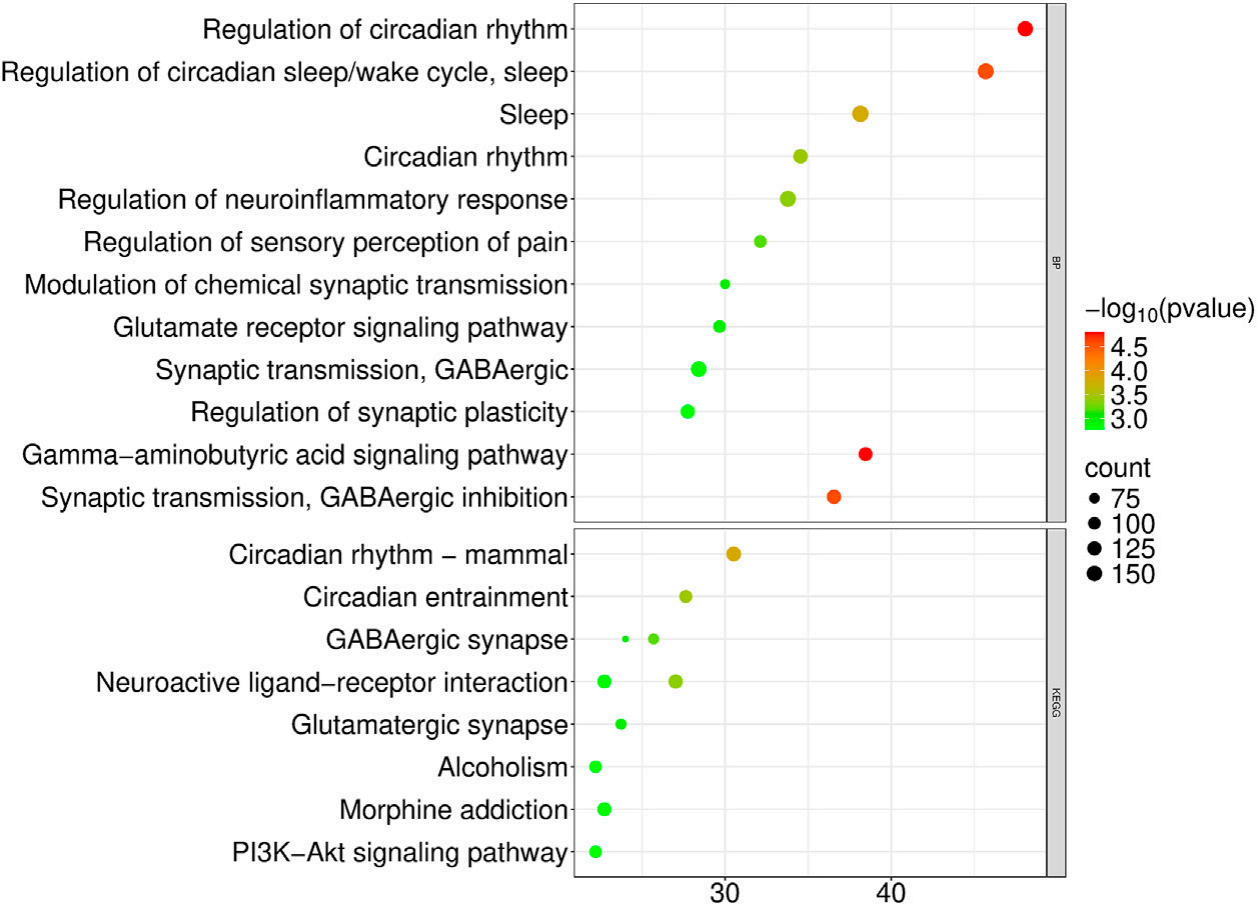
GO and KEGG analysis of the volatile components in the EVs from *P. orientalis* leaf.

The targets of the volatile components in EVs from *P. orientalis* leaf were identified using the Swiss Target Prediction, PubChem, TCMSP, and PharmMapper databases. Pathway enrichment analysis of these targets was performed using Metascape, with results selected under the criteria of p < 0.01 and enrichment score >20. The GO analysis revealed functions related to the regulation of circadian rhythm, regulation of the circadian sleep/wake cycle, sleep, and circadian rhythm (Figure 3). The KEGG analysis identified pathways associated with circadian rhythm in mammals, circadian entrainment, neuroactive ligand-receptor interactions, GABAergic synapses, and glutamatergic synapses (Figure 3). The primary functions of the volatile components in Platycladus orientalis-derived extracellular vesicles were linked to anxiety, depression, and insomnia. These findings are consistent with previous reports on the therapeutic effects of Platycladus orientalis, supporting its potential for treating anxiety, depression, and insomnia (Fan et al., 2012; Yan et al., 2022; Zheng et al., 2021).

### EVs from *P. orientalis* leaf were internalized by PC12 cells and attenuated corticosterone-induced neuronal damage and LDH release

**FIGURE 4:**
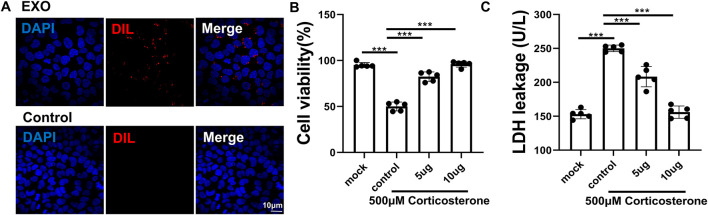
EVs from *P. orientalis* leaf attenuated corticosterone-induced neuronal damage and LDH release. **(A)** Laser confocal microscopy showed that Dil-labeled EVs entered the cytoplasm of PC16 cells. Scale bar, 10 μm. **(B)**The cell viability of PC16 cells treated with corticosterone (control), corticosterone plus 5 ug EVs (5 ug) or corticosterone plus 10ug EVs (10 ug). The PC16 cells without treatment served as a control (mock). **(C)** The LDH in the cell culture medium of PC16 cells treated with corticosterone (control), corticosterone plus 5 ug EVs (5ug) or corticosterone plus 10 ug EVs (10 ug). The PC16 cells without treatment served as a control (mock). n = 3 independent experiments. ns: p > 0.05; *: p < 0.05; **: p < 0.01; ***: p < 0.001.

DIL-labeled EVs from *P. orientalis* leaf were incubated with the rat adrenal medulla pheochromocytoma cell line PC12. Following incubation, the cells were washed three times with PBS and stained with DAPI. Microscopic analysis revealed numerous small, granular, DIL-labeled EVs (red) within the PC12 cells (blue) (Figure 4A), demonstrating the internalization of Platycladus orientalis-derived EVs by mammalian cells. Additionally, these EVs were found to reduce corticosterone-induced neuronal damage and LDH release in PC12 cells (Figures 4B,C).

### Anti-anxiety effect of EVs from *P. orientalis* leaf

**FIGURE 5:**
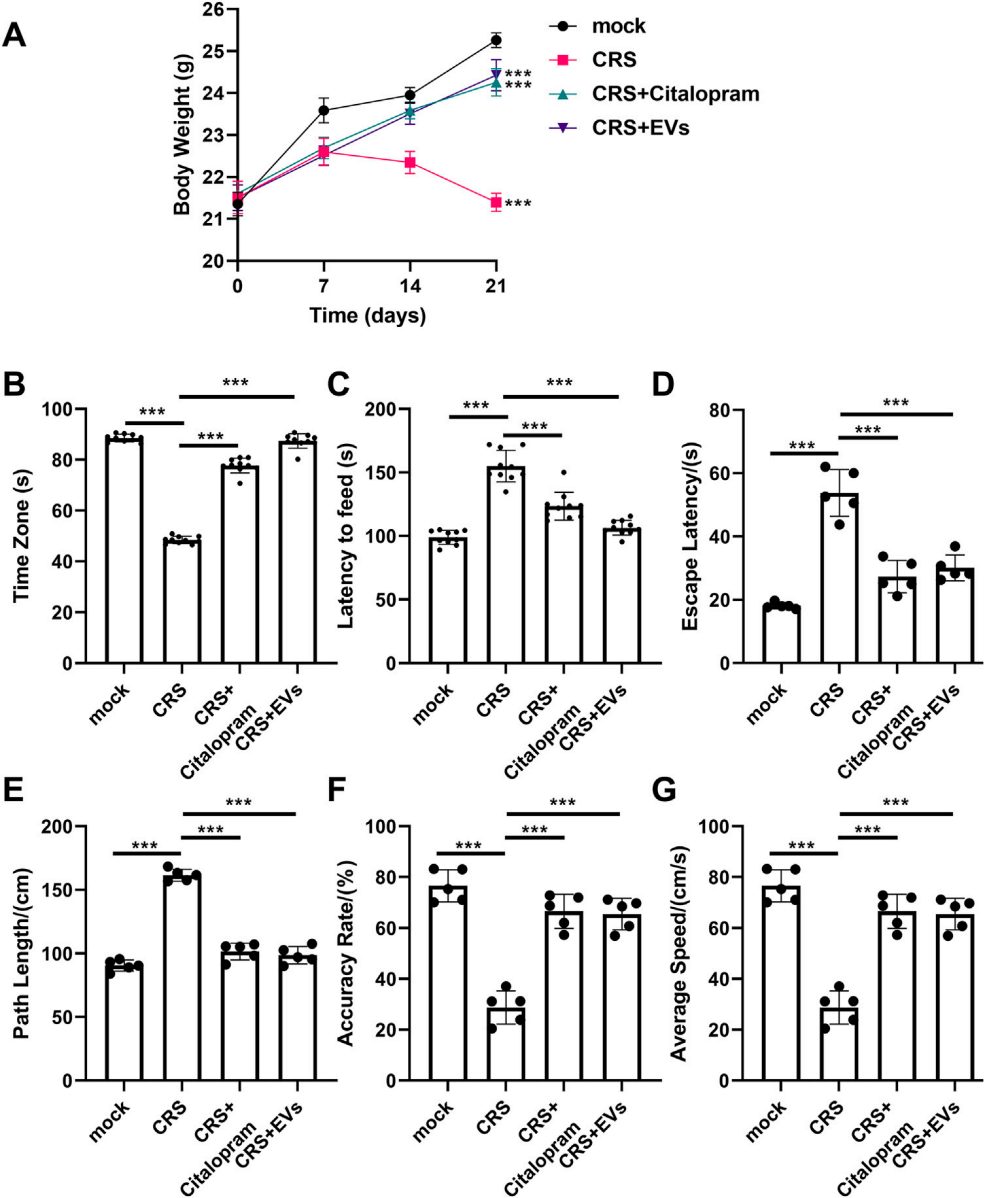
Anti-anxiety effect of EVs from *P. orientalis* leaf. **(A)** The body weight. **(B)** Th social interaction test (n = 10). **(C)** Novelty-suppressed feeding test (n = 10). **(D–G)** Morris water maze (MWM) test (5 mice was in per group). CRS mice treated with distilled water (CRS), citalopram (CRS + citalopram), or 10ug EVs (CRS + EVs). The mice without any treatment served as a control (mock). n = 3 independent experiments. ns: p > 0.05; *: p < 0.05; **: p < 0.01; ***: p < 0.001.

**FIGURE 6:**
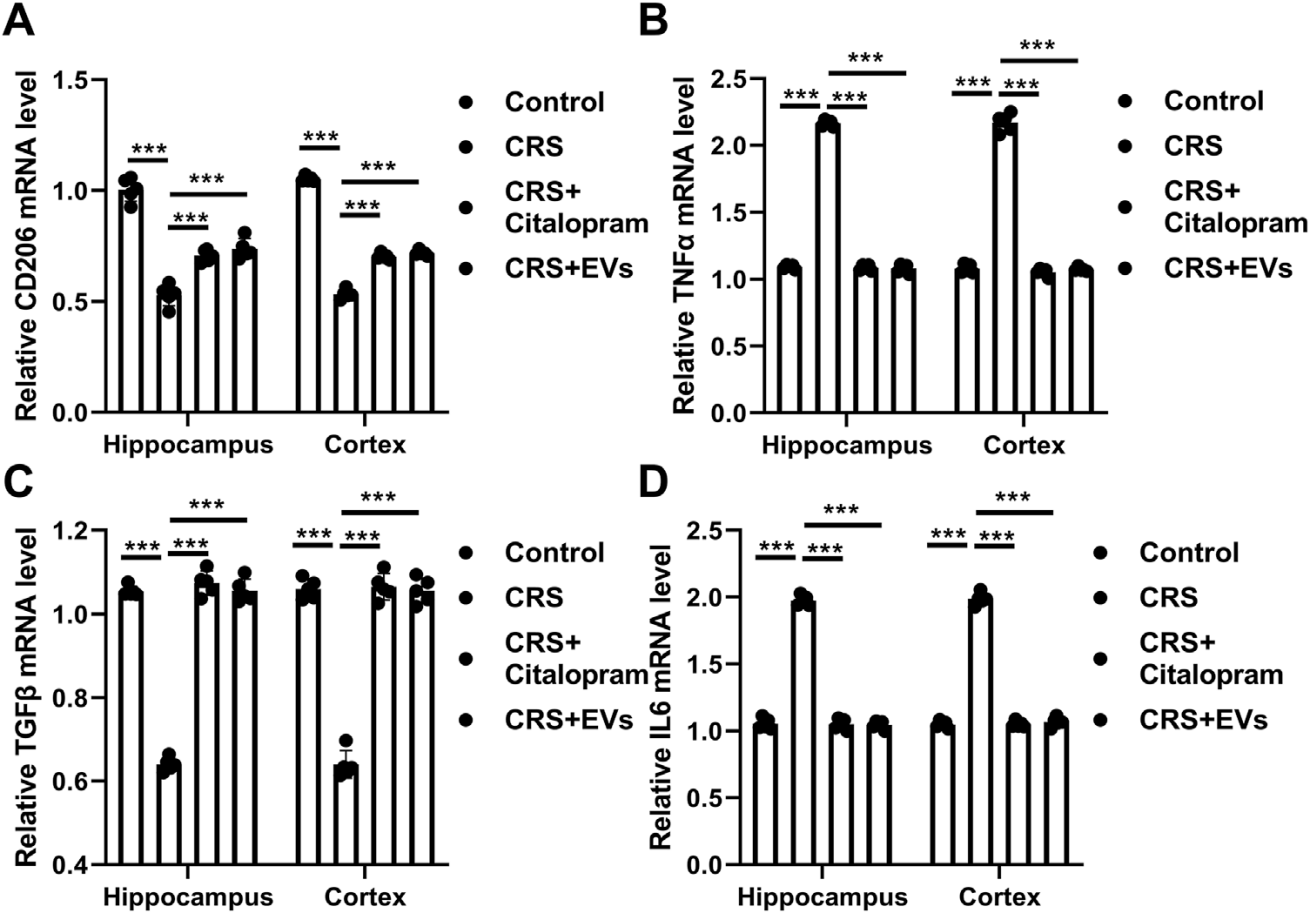
Effects of EVs from P. orientalis leaf on hippocampal and Cortex inflammatory cytokine CD206 **(A)**, TNFα **(B)**, TGFβ **(C)** and IL6 **(D)** mRNA expression in CRS mice. CRS mice treated with distilled water (CRS), citalopram (CRS+citalopram), or 10ug EVs (CRS+EVs). The mice without any treatment served as a control (mock). 5 mice was in per group. n = 3 independent experiments. ns: p > 0.05; *: p < 0.05; **: p < 0.01; ***: p < 0.001.

The CRS model induces anxiety-like behaviors, including social withdrawal and reduced body weight (Zhu et al., 2021). CRS mice exhibited decreased body weight and social interaction and increased feeding latency (Figures 5A–C). Treatment with P. orientalis-derived EVs significantly increased body weight, improved social interaction, and reduced feeding latency, comparable to escitalopram (10 mg/kg) (Figures 5A–C). Spatial learning and memory, assessed by the MWM, were impaired in CRS mice, as shown by increased escape latency and path length and decreased accuracy rate and average speed (Figures 5D–G). EV treatment reversed these deficits as the escitalopram (Figures 5D–G). Hippocampal and cortical expression of CD206, TNF-α, TGF-β, and IL-6 mRNA was normalized by EV treatment (Figures 6A–D), suggesting reduced neuroinflammation.

### Antidepressant effect of EVs from *P. orientalis* leaf

**FIGURE 7:**
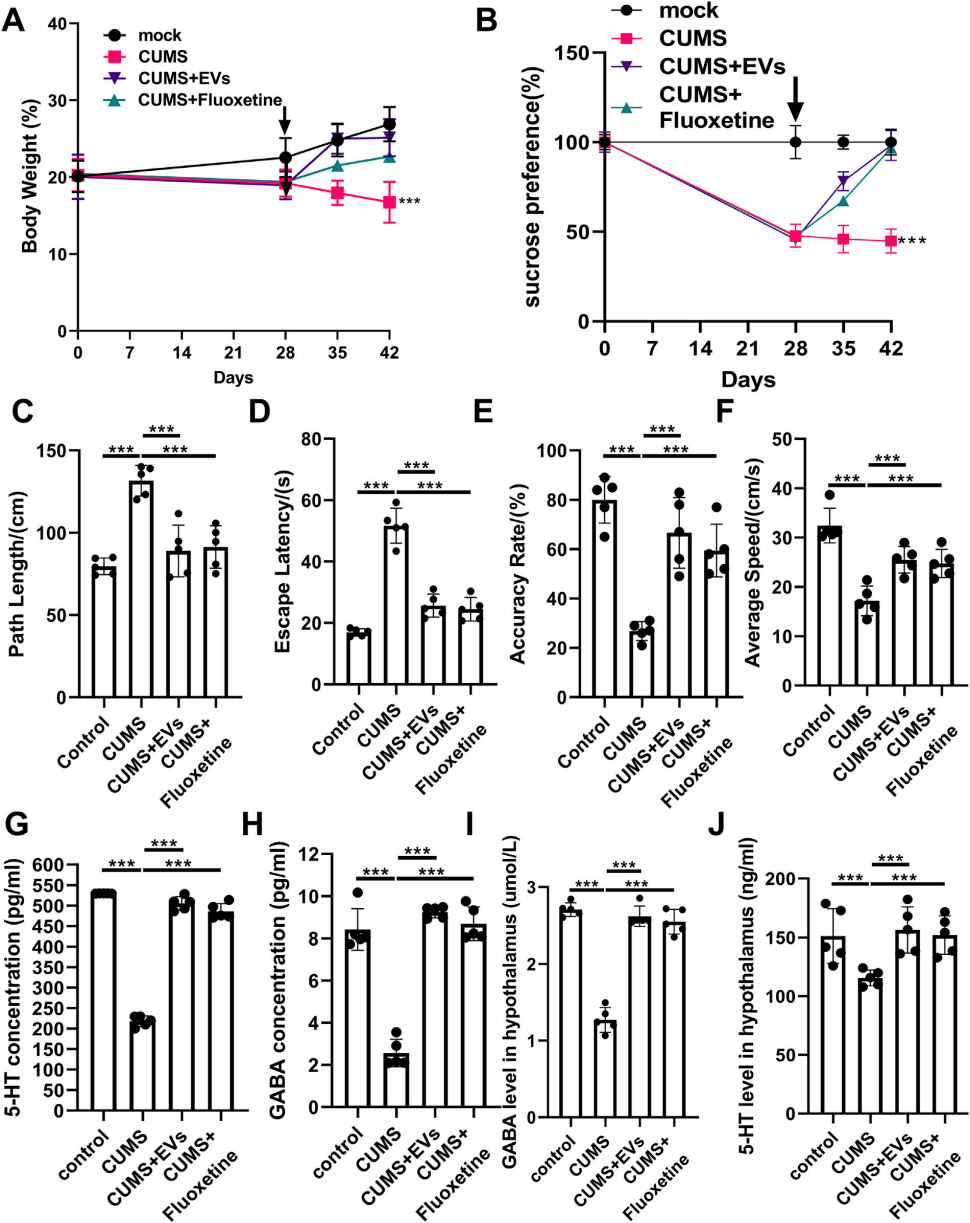
Antidepressant Effect of EVs from *P. orientalis* leaf. **(A)** The body weight. **(B)** Th sucrose preference test. **(C–F)** Morris water maze (MWM) test. **(G,H)** The concentration of 5-HT and GABA in the serum. **(I,J)** The concentration of 5-HT and GABA in the hippocampus. CUMS mice treated with distilled water (CUMS), Fluoxetine (CRS + Fluoxetine), or 10ug EVs (CUMS + EVs). The mice without any treatment served as a control (control). 5 mice was in per group. n = 3 independent experiments. ns: p > 0.05; *: p < 0.05; **: p < 0.01; ***: p < 0.001.

CUMS models depression-like phenotypes, including anhedonia and cognitive impairment (Yan et al., 2022). CUMS mice exhibited reduced body weight and sucrose preference, impaired MWM performance, and decreased serum and hippocampal 5-HT and GABA levels (Figures 7A–F). Intranasal EV treatment restored body weight, sucrose intake, spatial learning, and memory (Figures 7A–F), and increased 5-HT and GABA levels in both serum and hippocampus (Figures 7G–J), as the fluoxetine (a selective serotonin reuptake inhibitor used primarily to treat major depressive disorder). Notably, CRS and CUMS models exhibited overlapping behavioral deficits (reduced activity, impaired cognition) and neurochemical dysregulation (decreased 5-HT and GABA). While CRS primarily modeled anxiety-like behaviors and social deficits, CUMS primarily modeled depression-like behaviors (anhedonia, sucrose preference). EV treatment consistently improved both anxiety- and depression-related endpoints, highlighting their multimodal therapeutic potential across different stress-induced psychiatric phenotypes.

### Anti-Insomnia Effect of EVs from *P. orientalis* leaf

**FIGURE 8:**
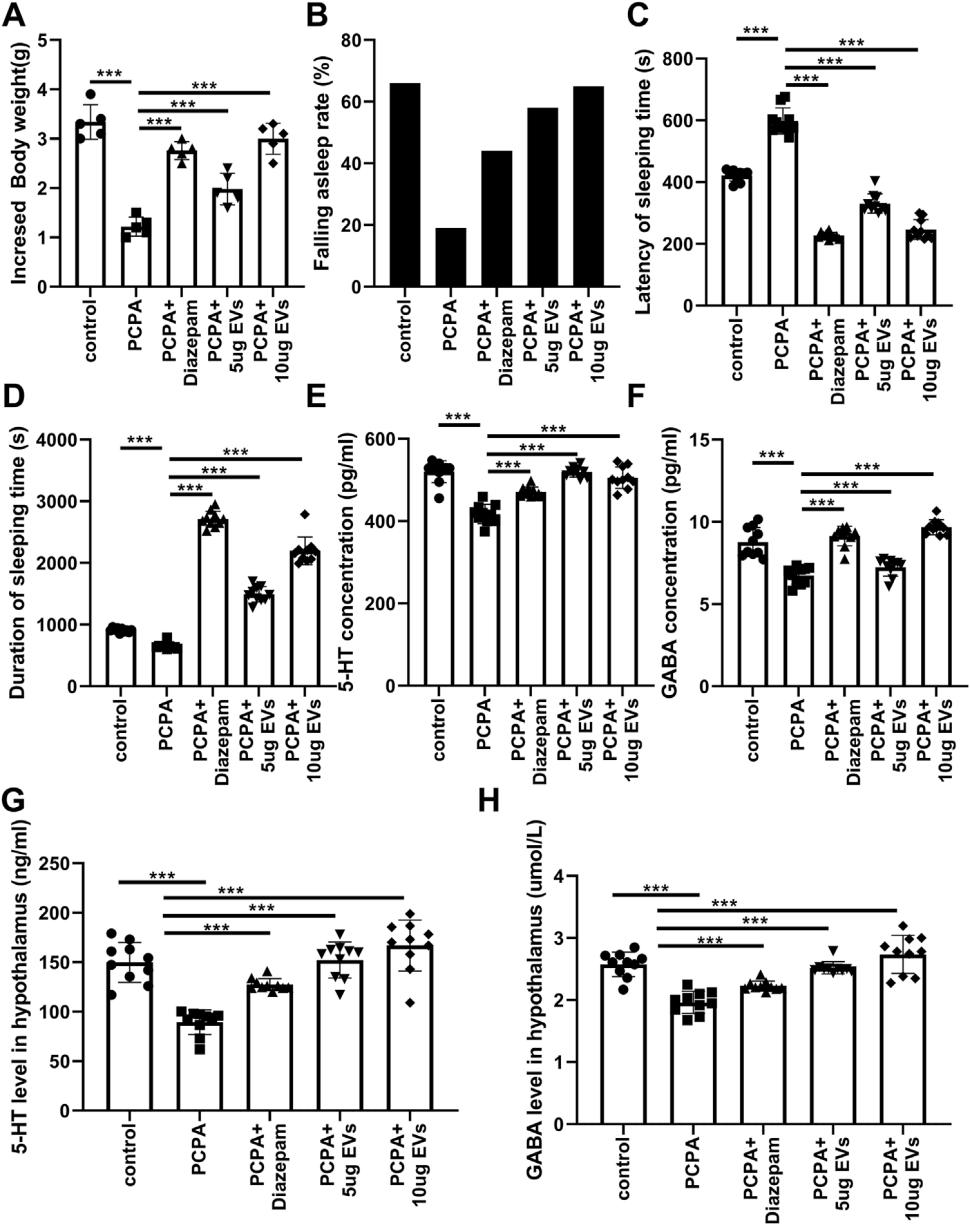
Anti-Insomnia Effect of EVs from *P. orientalis* leaf. **(A)** The body weight. **(B)** Th falling asleep rate. **(C,D)** The latency and duration of sleeping time. **(E,F)** The concentration of 5-HT and GABA in the serum. **(G,H)** The concentration of 5-HT and GABA in the hippocampus. PCPA treated mice treated with distilled water (PCPA), Diazepam (PCPA + Diazepam), 5 ug EVs (PCPA+ 5 ug EVs) or 10 ug EVs (CUMS+ 10 ug EVs). The mice without any treatment served as a control (control). 10 mice was in per group. n = 3 independent experiments. ns: p > 0.05; *: p < 0.05; **: p < 0.01; ***: p < 0.001.

PCPA is commonly used to treat carcinoid syndrome due to its ability to reduce serotonin (5-HT) levels in mammals. As a result, PCPA is widely used in research to induce insomnia in animal models, particularly in studies related to sleep disorders (Palagini et al., 2024; Guo et al., 2023). PCPA-induced insomnia decreased body weight, total sleep duration, and hippocampal and serum 5-HT/GABA levels (Figures 8A–H). While EV treatment significantly improved sleep onset, total sleep time, and neurotransmitter levels, comparable to diazepam (Figures 8A–H).

## Discussion

EVs mediate intercellular communication by delivering bioactive molecules, including lipids, proteins, and secondary metabolites (Buck and Nolte-'t Hoen, 2024). Plant-derived EVs offer additional advantages, including low immunogenicity and enrichment in compounds with anti-inflammatory, antioxidant, and neuroprotective properties (Zhu et al., 2024; Li et al., 2025; Nueraihemaiti et al., 2025; Zuo et al., 2025; Yu et al., 2025; Langellotto et al., 2025; Liu et al., 2024; Wang et al., 2025). Their therapeutic potential, particularly in the context of psychiatric disorders, is an emerging area of research. Anxiety, depression, and insomnia are among the most prevalent mental health conditions globally, with pathophysiology often involving dysregulation of neurotransmitter signaling, circadian rhythms, and neuroinflammation (Guo et al., 2023). Conventional treatments, such as selective serotonin reuptake inhibitors (SSRIs) and benzodiazepines, are limited by delayed onset, adverse effects, and risk of dependency. Thus, novel therapeutic strategies capable of modulating multiple biological pathways with minimal side effects are urgently needed. Plant-derived EVs, which can regulate neurotransmitter systems and attenuate neuroinflammation, represent a promising alternative for these disorders (Reed and Escayg, 2021; Yu et al., 2025; Wang et al., 2025).

*Platycladus orientalis (L.) Franco* has well-documented pharmacological activities, including anti-inflammatory, anxiolytic, and antidepressant effects (Fan et al., 2012; Mostafa et al., 2024; Korany et al., 2025; Darwish et al., 2022; Cui et al., 2023; Fan et al., 2011; Zhang et al., 2016; Ren et al., 2017; Lin et al., 2016; Ren et al., 2019a; Ren et al., 2019b; Gan et al., 2021). In our study, EVs derived from *P. orientalis* leaves exhibited significant therapeutic effects in rodent models of anxiety, depression, and insomnia. The CRS model primarily induced anxiety-like behaviors, such as social withdrawal and altered feeding, whereas the CUMS model mainly caused depression-like phenotypes, including anhedonia and cognitive deficits. EV treatment improved both anxiety- and depression-related behaviors, demonstrating multimodal efficacy. In the PCPA-induced insomnia model, EV administration restored sleep parameters and normalized neurotransmitter levels. These findings are consistent with prior studies reporting anxiolytic and antidepressant effects of *P. orientalis* extracts (Fan et al., 2012; Mostafa et al., 2024; Ren et al., 2019b; Yan et al., 2022; Xu C. et al., 2024; Zhou et al., 2023; Yu et al., 2020; Liang et al., 2023; Liu et al., 2020; Feng et al., 2022; Jing et al., 2016; Zhao et al., 2025; Guo et al., 2021; Cho et al., 2025; Liu et al., 2013; Bae et al., 2021; Kim et al., 2024; Zhao et al., 2024; Yan et al., 2020; Chang et al., 2012; Liao et al., 2024; Wu et al., 2023) and extend them by showing enhanced efficacy through EV encapsulation, which may improve bioavailability and CNS targeting. Mechanistically, EV treatment restored serum and hippocampal levels of serotonin (5-HT) and γ-aminobutyric acid (GABA), reduced pro-inflammatory markers (TNF-α, IL-6), and increased anti-inflammatory TGF-β expression. This dual modulation of neurotransmission and neuroinflammation aligns with bioinformatic predictions of EV-associated volatile compounds affecting circadian rhythm and GABAergic/glutamatergic synapses, supporting a mechanistic link between EV cargo and therapeutic outcomes.

Our findings extend the growing body of research on plant-derived extracellular vesicles by demonstrating their efficacy in stress-related psychiatric and sleep disorders. Prior work with plant derived EVs has largely focused on peripheral models, such as inflammation, intestinal injury, and cancer, using oral or systemic administration routes (Zhu et al., 2024; Li et al., 2025; Nueraihemaiti et al., 2025; Zuo et al., 2025; Yu et al., 2025; Langellotto et al., 2025; Liu et al., 2024; Wang et al., 2025; Xu et al., 2025). For example, ginseng-derived EVs have been shown to promote healing of infected wounds (Ye et al., 2025), and EVs derived from edible plants have been emphasized for their extensive range of physiological regulatory functions (Yang et al., 2023). However, these studies did not evaluate CNS-directed effects. Our study showed intranasal administration of EVs from *P. orientalis* leaf produces robust antidepressant-, anxiolytic-, and soporific-like effects across three complementary rodent models, an approach that enhances translational relevance by exploiting the nose-to-brain route to bypass the BBB. We also systematically link behavioral improvements to restoration of 5-HT and GABA levels, reduction of pro-inflammatory cytokines (IL-6, TNF-α), and the presence of bioactive volatile compounds identified by GC–MS. These results suggest that EVs from *P. orientalis* leaf act through multimodal pathways involving neurotransmitter regulation and neuroinflammation suppression.

Across CRS, CUMS, and PCPA models, EVs consistently restored neurochemical balance and reduced neuroinflammatory markers. Although CRS and CUMS elicited distinct behavioral phenotypes (anxiety versus depression), the overlapping neurochemical and molecular dysregulations suggest that *P. orientalis* leaf-derived EVs exert broad multimodal effects relevant to multiple psychiatric conditions. Compared with other plant-derived EVs, such as those from ginger and grape, which modulate inflammation and neurotransmitter systems (Yu et al., 2025), the EVs from *P. orientalis* leaf demonstrated comparable or stronger effects across multiple psychiatric models, highlighting their translational potential.

Overall, our findings support the therapeutic potential of EVs from *P. orientalis* leaf as a novel approach for managing anxiety, depression, and insomnia. Encapsulation of bioactive compounds within EVs may enhance targeted delivery, bioavailability, and efficacy, providing a promising alternative to conventional pharmacotherapies. Future research should focus on detailed molecular characterization of EV cargo, elucidation of underlying mechanisms, and clinical studies to evaluate safety and efficacy in humans, paving the way for potential plant-EV-based therapies alone or in combination with existing treatments.

## Limitations

While the findings of this study are promising, several limitations and gaps should be acknowledged. First, the characterization of EVs from *P. orientalis* leaf remains challenging, as their molecular cargo can vary depending on plant species, environmental conditions, and extraction methods. Although this study identified the major bioactive compounds in EVs from *P. orientalis* leaf, the full molecular profile of these vesicles remains incompletely characterized. Further investigation is needed to determine the specific components responsible for their therapeutic effects, including lipids, proteins, and RNA species. Second, this study relied on rodent models, which, while valuable for simulating human psychiatric conditions, cannot fully recapitulate the complexity of human neurobiology. Species-specific differences in neurotransmitter pathways, immune responses, and behavior may limit the direct translatability of these results. Moreover, the current study focused on the acute therapeutic effects of EVs from *P. orientalis* leaf; their long-term safety and efficacy remain unexplored. Extended administration studies in animal models, as well as clinical trials, will be essential to assess potential chronic benefits and any associated adverse effects. Third, this study primarily employed behavioral and biochemical assessments to evaluate the effects of EVs on neurochemistry and mental health. While our results provide preliminary mechanistic insights—demonstrating that EV treatment restored 5-HT and GABA levels in serum and brain tissue and reduced neuroinflammatory markers such as TNF-α, IL-6, and TGF-β—further research is required to elucidate the precise molecular mechanisms by which EVs from *P. orientalis* leaf interact with neuronal signaling networks. In particular, a detailed characterization of EV cargo, including lipids, proteins, and small RNAs, and its influence on neurotransmitter regulation and neuroinflammatory pathways will be critical for fully understanding the therapeutic potential of plant-derived EVs in psychiatric disorders. Forth. we did not perform *in vivo* biodistribution analyses after intranasal delivery due to serveral technical shortcomings, which are essential to confirm brain localization.

## Conclusion

In summary, our study demonstrates that EVs from *P. orientalis* leaf exert significant therapeutic effects in rodent models of anxiety, depression, and insomnia. EV treatment improved behavioral deficits across CRS, CUMS, and PCPA-induced insomnia models, restored key neurotransmitter levels (5-HT and GABA) in serum and brain, and modulated neuroinflammatory markers, including TNF-α, IL-6, and TGF-β. These results highlight the multimodal mechanism of EVs from *P. orientalis* leaf, which combine neurochemical regulation with anti-inflammatory effects. Compared with other plant-derived EVs, EVs from *P. orientalis* leaf showed robust efficacy across multiple psychiatric phenotypes, supporting their translational potential as a novel, plant-based therapeutic strategy. Future studies should focus on comprehensive molecular characterization of EV cargo and clinical evaluation to assess safety, efficacy, and therapeutic applicability in human populations.

## Data Availability

The original contributions presented in the study are included in the article/Supplementary Material, further inquiries can be directed to the corresponding authors.
