# Supplementary material for: Therapeutic potential of extracellular vesicles derived from Platycladus Orientalis leaf in treating anxiety, depression, and insomnia
Source: Front Pharmacol. 2025 Sep 29;16:1693106. doi: 10.3389/fphar.2025.1693106 (PMC12515933; doi:10.3389/fphar.2025.1693106)
Supplement: Supplementary file 1 [file Supplementaryfile1.docx]

**Table S1. The primers for RT-qPCR.**

|  | **Forward** | **Reverse** |
| --- | --- | --- |
| **CD206** | CTCTGTTCAGCTATTGGACGC | CGGAATTTCTGGGATTCAGCTTC |
| **TNFα** | CCCTCACACTCAGATCATCTTCT | GCTACGACGTGGGCTACAG |
| **TGFβ** | CTCCCGTGGCTTCTAGTGC | GCCTTAGTTTGGACAGGATCTG |
| **IL6** | TAGTCCTTCCTACCCCAATTTCC | TTGGTCCTTAGCCACTCCTTC |
| **Gapdh** | AGGTCGGTGTGAACGGATTTG | GGGGTCGTTGATGGCAACA |

**Table S2. Volatile components in the EVs from P. orientalis leaf .**

| **Number** | **RetentionIndex** | **kind** | **compound** | **Intensity** |
| --- | --- | --- | --- | --- |
| **30** | 1333 | sh | δ-elemene | 0.228571429 |
| **31** | 1342 | sh | (+)-α-longipinene | 1.152380952 |
| **32** | 1338 | sh | α-cubebene | 0.038095238 |
| **33** | 1370 | sh | α-copaene | 0.80952381 |
| **34** | 1387 | sh | β-elemene | 0.466666667 |
| **35** | 1411 | sh | β-caryophyllene | 1.057142857 |
| **36** | 1419 | sh | (-)-thujopsene | 0.114285714 |
| **37** | 1428 | sh | elixene | 1.4 |
| **38** | 1442 | sh | cis-muurola-3,5-diene | 0.104761905 |
| **39** | 1446 | sh | α-humulene | 0.047619048 |
| **40** | 1473 | sh | germacreneD | 0.40952381 |
| **41** | 1478 | sh | α-curcumene | 0.123809524 |
| **42** | 1484 | sh | cis-muurola-4(15),5-diene | 0.114285714 |
| **43** | 1493 | sh | α-muurolene | 0.304761905 |
| **44** | 1507 | sh | γ-muurolene | 0.19047619 |
| **45** | 1510 | sh | γ-cadinene | 0.647619048 |
| **46** | 1515 | sh | (15,25,4R)-(-)-α-α-dimethyl-1-vinyl-o-menth-8-ene-4-methanol | 0.133333333 |
| **47** | 1518 | sh | δ-cadinene | 0.238095238 |
| **48** | 1543 | sh | (R)-β-himachalene | 0.123809524 |
| **50** | 1548 | sh | germacreneB | 1.79047619 |
| **49** | 1545 | os | elemol | 8.876190476 |
| **51** | 1568 | os | germacreneD-4-ol | 0.342857143 |
| **52** | 1574 | os | caryophylleneoxide | 0.142857143 |
| **53** | 1587 | os | α⁃cedrol | 0.114285714 |
| **54** | 1621 | os | (+)-epicubenol | 1.533333333 |
| **55** | 1624 | os | γ-eudesmol | 1.180952381 |
| **56** | 1631 | os | hinesol | 0.076190476 |
| **57** | 1641 | os | β-eudesmol | 1.942857143 |
| **58** | 1645 | os | α-eudesmol | 1.876190476 |
| **59** | 1659 | os | bulnesol | 0.076190476 |
| **60** | 1667 | os | bisabolol | 0.057142857 |
| **15** | 1063 | om | cis-sabinenehydrate | 0.114285714 |
| **17** | 1093 | om | trans-sabinenehydrate | 0.085714286 |
| **20** | 1130 | om | cis-(-)-limoneneoxide | 0.219047619 |
| **21** | 1156 | om | sabinaketone | 0.133333333 |
| **22** | 1172 | om | (-)-terpinen-4-ol | 0.057142857 |
| **23** | 1242 | om | thymolmethylether | 0.161904762 |
| **24** | 1250 | om | γ-terpineol | 0.076190476 |
| **25** | 1255 | om | linalylacetate | 0.066666667 |
| **26** | 1261 | om | methylcitronellate | 0.180952381 |
| **27** | 1265 | om | phellandral | 0.447619048 |
| **28** | 1281 | om | bornylacetate | 0.228571429 |
| **29** | 1296 | om | 4-terpinenylacetate | 0.038095238 |
| **61** | 2045 | od | manool | 1.723809524 |
| **62** | 2274 | od | trans-totarol | 0.247619048 |
| **1** | 923 | mh | α-thujene | 1.038095238 |
| **2** | 930 | mh | α-pinene | 42.39047619 |
| **3** | 942 | mh | camphene | 0.361904762 |
| **4** | 964 | mh | sabinene | 0.142857143 |
| **5** | 969 | mh | β-pinene | 11.77142857 |
| **6** | 990 | mh | β-myrcene | 1.780952381 |
| **7** | 996 | mh | δ-2-carene | 0.933333333 |
| **8** | 1000 | mh | α-phellandrene | 0.076190476 |
| **9** | 1006 | mh | p-mentha-1(7),8-diene | 2.657142857 |
| **10** | 1012 | mh | α-terpinene | 0.095238095 |
| **11** | 1018 | mh | 1,3,8-p-menthatriene | 0.028571429 |
| **12** | 1020 | mh | p-cymene | 0.123809524 |
| **13** | 1025 | mh | d-limonene | 9.085714286 |
| **14** | 1055 | mh | γ-terpinene | 0.114285714 |
| **16** | 1084 | mh | terpinolene | 0.171428571 |
| **18** | 1106 | ie | isopentylisovalerate | 0.057142857 |
| **19** | 1117 | ie | 3-methylbut-3-enylisovalerate | 0.057142857 |

**Figure s1 Effect of the EVs from P. orientalis leaf on immobility time in tail suspension of CUMS mice model.**
